# A randomized double-blind, placebo-controlled, cross-over trial assessing the effect of tadalafil (Cialis) on the cardiovascular response in men with complete spinal cord injury above the sixth thoracic level: A Pilot Study

**DOI:** 10.1038/s41394-018-0137-9

**Published:** 2018-11-21

**Authors:** Karen D. Ethans, Alan Casey, Mohamed Tarhoni, Mayur Nankar, Stella Entcheva

**Affiliations:** 1 University of Manitoba, Health Sciences Centre, Winnipeg, MB Canada; 2 Leonard A. Miller Centre St. John’s NFLD, St. John’s, Canada

## Abstract

**Study design:**

Double-blind, randomized cross-over placebo-controlled pilot study.

**Objectives:**

To determine the effects of tadalafil on systolic blood pressure (SBP), heart rate (HR), and dizziness of men with American Spinal Injury Association Impairment Scale-A (AIS-A) spinal cord injury (SCI) between cervical-4 (C4) and thoracic-5 (T5) levels.

**Setting:**

Outpatient rehabilitation clinic.

**Design:**

Double-blind, randomized cross-over placebo-controlled pilot study.

**Methods:**

20 males with AIS-A SCI, C4-T5 received either tadalafil 20 mg or placebo for the first arm, and then were crossed-over after 1 week to the second arm. SBP, HR, and Visual Analogue Scale (VAS) for dizziness upon sitting up from lying were measured at baseline and again 1, 2, 4, 12, 22, 29, and 36 h post dose administration. The change in each outcome measure (SBP, HR, VAS dizziness) was observed from pre-dose to each time point. A change in VAS dizziness of 2 cm or greater (scale 0–10 cm) was considered positive.

**Results:**

SBP did not change significantly in either group. However, HR increased significantly in the tadalafil group at several time points (12 h *p* < 0.05, 22 h *p* <0.05, 29 h *p* <0.01, and 36 h *p* <0.05), with no change in the placebo group. The VAS dizziness significantly increased (range 2–6 cm changes) at some time point in 1/4 of the subjects after tadalafil, but not in the placebo group; all reports of dizziness were at 12 h or later.

**Conclusions:**

Tadalafil use in people with SCI above T6 is safe with respect to not causing hypotension; hemodynamic changes that occurred 12–36 h post administration were compensated for by elevations in HR.

**Sponsorship:**

The Manitoba Medical Services Foundation and the Health Sciences Centre Foundation.

## Introduction

Erectile dysfunction (ED) is the persistent inability to achieve or maintain an erection sufficient for satisfactory sexual intercourse [1], and is a common issue among males with spinal cord injury (SCI) [2]. Young men comprise the largest population of patients with SCI (60% are 16–30 years old) and males (80%) [3]. Eighty percent of patients with SCI achieve some type of erection, either reflexogenic, psychogenic, or mixed, but is rarely sufficient to have sexual intercourse without medications [4].

The first-line of treatment for ED in men with SCI is oral phosphodiesterase type-5 (PDE-5) inhibitors [5, 6]. The mechanism of action of these medications involves active inhibition of the PDE-5 enzyme, resulting in an increase in cyclic guanosine mono phosphate with subsequent smooth muscle relaxation of the corpora cavernosa of the penis, allowing for blood to enter and erection to occur when stimulation occurs [5]. Sildenafil (Viagra)^TM^ was the first PDE-5 inhibitor available and more recently available are tadalafil (Cialis)^TM^ [7], vardenafil (Levitra)^TM^, and others. All of these drugs appear to be generally safe and well tolerated, with similar side effects [8, 9]. Sildenafil has a half-life of about 4 h [10], whereas tadalafil has a mean half-life of 17.5 h [11]. With adequate sexual stimulation, significant erectile response has been observed as early as 16 min and as long as 36 h after dosing with tadalafil in about 50% of men with ED [12]. Sildenafil, tadalafil, and vardenafil all have proven efficacy in the literature in men with SCI [13–24]. Tadalafil has been shown to improve erectile function over baseline and compared to placebo regardless of the American Spinal Injury Association Impairment Score (AIS) [21]. The improved erectile function experienced with tadalafil in men with SCI has been shown to continue 12–24 h post-dosing [16, 21, 22]. However, despite of all the efficacy studies in men with SCI using tadalafil, no studies have been done to measure cardiovascular responses in this population after dosing with tadalafil.

People with SCI are at risk of developing orthostatic hypotension, especially those with lesions at or above T5, due to decreased resting levels of circulating catecholamines [25, 26], and no significant release of epinephrine or norepinephrine when changing from a lying to a sitting position. The main concern in this population would be the orthostatic hypotension being caused or exacerbated by PDE-5 inhibitors. An earlier study by our group of the shorter-acting PDE-5 inhibitor, sildenafil, revealed that it causes orthostatic hypotension, tachycardia, and dizziness after administration in the SCI population, particularly in those with tetraplegia, and suggested that caution should be used when prescribing sildenafil to persons with SCIs, as blood pressure can drop significantly [27]. Sipski et al. reported similar results, although only trending to significance, likely a false negative due to small sample size [28]. However, due to the longer lasting effect and longer half-life of tadalafil, there is a concern that any side effects may last longer as well. This has recently been confirmed in a study of men that were not spinal cord injured, where it was found that although the side effect prevalence was similar with the various PDE-5 inhibitors, the duration of the side effects was significantly longer with tadalafil (14.9 h) vs. sildenafil (3.9 h) [29].

Therefore, our primary objective in the present study is to assess the effects of administrating tadalafil 20 mg compared to placebo on the systolic blood pressure (SBP) and heart rate (HR) of men with complete American Spinal Injury Association Impairment Scale-A (AIS-A) SCI above the sixth thoracic level (T6), and how long these effects last. The secondary objective is to compare adverse events with the administration of one dose of tadalafil 20 mg vs. placebo, with measuring dizziness on a Visual Analog Scale (VAS) as an adverse event. As there are several previous studies measuring the efficacy of tadalafil in this population, this aspect was not felt to be a knowledge gap, and therefore efficacy of this medication in the SCI population was not studied again in this particular safety study.

## Methods

This was a prospective, randomized, double-blind, placebo controlled, cross-over study of male subjects with AIS-A SCI between the fourth cervical (C4) and fifth thoracic (T5) levels. Subjects enrolled had to be 18–70 years old, a minimum of 6 months post SCI, and able and willing to consent to participate. Excluded were subjects who were diabetic, taking nitroglycerin in any form, had ischemic heart disease or a significantly abnormal electrocardiogram, had lower motor neuron dysfunction, were heroin or cocaine users, had a history of adverse reactions to tadalafil or any other PDE-5 inhibitor, or had used any other PDE-5 inhibitor medications within a week before administration of the study medications. This study was approved by the University of Manitoba Research and Ethics board and registered with Clinicaltrials.gov (registration number NCT01067391).

Subjects were randomly assigned to one of two arms in a double-blinded fashion by our pharmacy. Prior to checking these measures the subject was lying flat for at least 10 min, then sat up and measures were done within 1 min. Those assigned to arm 1 received tadalafil first, arm 2 received placebo first. SBP, HR, and dizziness on VAS (range 0–10 cm) were measured. The pill was then given (tadalafil for arm 1, placebo to arm 2), and the measurements repeated hourly for 2 h, then 4 h post-dose.

After the 4 h measurement, the subject went home and repeated all these measures with an automated BP and HR apparatus at 12 h, 22 h, then every 7 h × 2 (to 36 h post-dose). One week later, the subject returned and was crossed-over to the other arm, with the above procedures and measures repeated.

All applicable institutional regulations concerning the ethical use of human volunteers were followed during the course of this research.

### Statistical analyses

Sample size calculation was done using G* power software showed that for a moderate effect size 0.5 at 20% type 2 error rate we need to recruit *n* = 32 participants. The change in each outcome measure (SBP, HR, VAS) was observed with effects of time from pre-dose to each time point. Linear mixed model was used to test our hypothesis and analyze outcomes using SAS program. A change at any time point in the VAS of 2 or greater was considered positive. Significant comparison was made using post hoc pairwise corrections. The level of significance was *α* < 0.05.

## Results

All 20 participants who were enrolled successfully completed the study (Table 1).

**Table 1:** Baseline characteristics

*N*	20
Age (mean with range)	46 (29–68) years
Chronicity of injury (mean with range)	17.7 (1–41) years
NLI cervical (C4–C8)	*n* = 12
NLI thoracic (T1–T5)	*n* = 8

*NLI* neurological level of injury

**Fig. 1:**
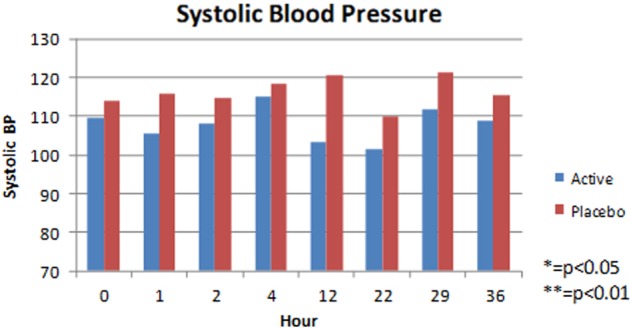
Mean systolic blood pressure, at each time point, in sitting position

**Fig. 2:**
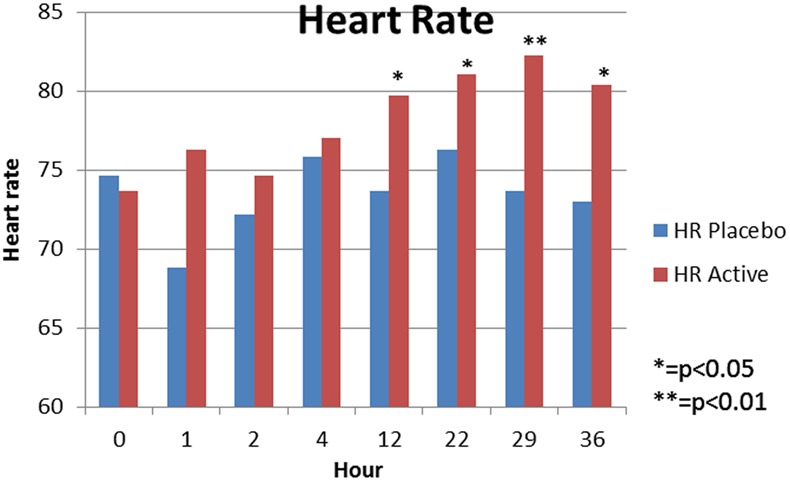
Mean heart rate at each time point, in sitting position

SBP did not change from baseline significantly in either group at any time point. (Fig. 1). Sub-analysis of the cervical level group against the thoracic level group also revealed no significant SBP change at each time point. The HR, however, was increased significantly in the tadalafil group at several time points (12 h *p* < 0.05, 22 h *p* < 0.05, 29 h *p* < 0.01, and 36 h *p* < 0.05) compared to baseline, with no change in the placebo group (Fig. 2).

Based on the predefined clinical significance of two points on the VAS for dizziness, there was a significant dizziness at some time point in 1/4 of the subjects after administration of tadalafil, but there were none that reported any dizziness in the placebo group. All reports of dizziness in the active group were at 12 h or later. The scores were low 2/10 at three time points for one subject, 3/10 at one time point for another subject, 3/10 and 6/10 at one time point each for a third subject, and 6/10 at one time point for the fourth subject that reported any dizziness. 3/4 of the participants who reported dizziness had cervical level lesions; one had a thoracic level lesion.

Participants reported dizziness on the VAS, but none of them complained about dizziness being a problem. No other adverse events were noted during the trial.

## Discussion

PDE-5 inhibitors are the preferred treatment for ED [30], and there has been proven efficacy of these medications in men with SCI. Those with SCI, especially those with lesions above T6, are at great risk for hypotension, and sildenafil, can exacerbate this hypotension. Yet previously we had no such safety parameters for the longer-acting tadalafil, despite the potential risk for hypotension lasting up to 36 h.

Our study found that there is no significant drop in SBP after using tadalafil compared to placebo. However, there was a significant increase in HR at all-time points 12 h and greater in the tadalafil group, with no change in the placebo group. This suggests that although SBP did not drop, the medication causes a reduction in peripheral vascular tone, and that HR must increase in a compensatory manner in order to maintain cardiac output and hence SBP. Post hoc analysis of the cervical (C4–C8) and thoracic level (T1–T5) groups did not change our results as SBP did not change significantly in either group. HR was significantly increased in some time points in both groups in the post hoc analysis but only “trended” to increase in some time points. We had small numbers in each of these sub-groups and thus cannot draw any different conclusions from this post hoc analysis other than it appears that the response was not different between the levels. Perhaps, if there were a larger sample size this comparison would have been more valid to assess the effect of some sympathetic chain innervation (which starts at T1). In our previous study on sildenafil [27], we did find that those with C8 and above lesions had lowering of SBP, whereas those with T1–T5 lesions did not, but had HR elevations, presumably due to the ability of the subjects T1–T5 to have compensatory HR increase [25].

We only performed measurements up to 36 h post-administration, which, in hindsight, was not long enough, given that we found significant changes up to and including the 36 h point. Although we counsel patients the therapeutic window of tadalafil is up to 36 h, we also counsel not to repeat a dose within 72 h, which, given our findings, is likely appropriate, as we suspect that the hemodynamic effects may last longer than 36 h.

This was a cross-over study, thus many confounding factors were eliminated as subjects acted as their own control. However, we recognize that with the small sample size, type-2 error may have occurred; we may have achieved false negative results with respect to lack of SBP drop. In analyzing the data, however, there was little to no trend to suggest increased numbers may have led to significance. Physiologically, it would be expected that the cervical spinal injured (tetraplegic) AIS-A men would not be able to compensate for peripheral vasodilation by increasing heart-rate, although the small sub-analysis of this group did not show drop in SBP and did show higher HR. Despite this finding, it would be prudent to further study just cervical level injured men in a larger sample size to test this further and avoid type-2 error.

We found a clinically significant change (>2 at any time point) in the amount of dizziness on a VAS scale with active medication at time points 12 h and greater, which corresponds to the timing of the compensatory HR response seen at all time points 12 h and greater. Therefore, although the SBP was not affected, findings suggest that some of the subjects have perception of the hemodynamic changes that occur after tadalafil use. Aside from the rating of dizziness, no subjects stated dizziness was problematic, and no other adverse events were noted. Overall, tadalafil was tolerated by all of the study participants and hence likely to be considered a safe and efficacious alternative as indicated in different studies [16, 22, 23].

## Conclusions

The use of tadalafil in people with SCI lesions above T6 is safe with respect to not causing hypotension; hemodynamic changes occur 12–36 post-administration are compensated for by elevations in HR to maintain cardiac output. However, this should be further examined in a study with a larger sample size. Care and caution should still be taken, especially in the tetraplegic population, as those without spinal cord sympathetic outflow (C8 and above) may not be able to have adaptations in HR to maintain blood pressure.

## References

[1] National Institutes of Health. (1993). Consensus development panel on impotence. NIH Consensus Conference: Impotence. J Am Med Assoc.

[2] Smith EM, Bodner DR (1993). Sexual dysfunction after spinal cord injury. Urol Clin North Am.

[3] National Spinal Cord Injury Statistical Center. Facts and figures at a glance. Birmingham, AL: University of Alabama at Birmingham; 2016. p. 1–2.

[4] Courtois F, Charvier K, Leriche A, Raymond D, Eyssette M (1995). Clinical approach to erectile dysfunction in spinal cord injured men. A review of clinical and experimental data. Spinal Cord.

[5] Ramos A, Samso J (2004). Specific aspects of erectile dysfunction in spinal cord injury. Int J Impot Res.

[6] Lombardi G, Musco S, Kessler TM, Li Marzi V, Lanciotti M, Del Popolo G (2015). Management of sexual dysfunction due to central nervous system disorders: a systematic review. BJU Int.

[7] Boolell M, Allen MJ, Ballard SA, Gepi-Attee S, Muirhead GJ, Naylor AM (1996). Sildenafil: an orally active type 5 cyclic GMP-specific phosphodiesterase inhibitor for the treatment of penile erectile dysfunction. Int J Impot Res.

[8] Rosen RC, McKenna KE (2002). PDE-5 inhibition and sexual response: pharmacological mechanisms and clinical outcomes. Annu Rev Sex Res.

[9] Brock GB, McMahon CG, Chen K, Costigan T, Shen W, Watkins V (2002). Efficacy and safety of tadalafil for the treatment of erectile dysfunction: results of integrated analyses. J Urol.

[10] Nichols DJ, Muirhead GJ, Harness JA (2002). Pharmacokinetics of sildenafil after single oral doses in healthy male subjects: absolute bioavailability, food effects and dose proportionality. Br J Clin Pharmacol.

[11] Forgue ST, Patterson BE, Bedding AW, Payne CD, Phillips DL, Wrishko RE (2006). Tadalafil pharmacokinetics in healthy subjects. Br J Clin Pharmacol.

[12] Patterson B, Bedding A, Jewell H, Payne C, Mitchell M (2001). The effect of intrinsic and extrinsic factors on the pharmacokinetic properties of tadalafil (IC351). Int J Impot Res.

[13] Schmid DM, Schurch B, Hauri D (2000). Sildenafil in the treatment of sexual dysfunction in spinal cord-injured male patients. Eur Urol.

[14] Hultling C, Giuliano F, Quirk F, Pena B, Mishra A, Smith M (2000). Quality of life in patients with spinal cord injury receiving VIAGRA®(sildenafil citrate) for the treatment of erectile dysfunction. Spinal Cord.

[15] Derry F, Hultling C, Seftel AD, Sipski ML (2002). Efficacy and safety of sildenafil citrate (Viagra®) in men with erectile dysfunction and spinal cord injury: a review. Urology.

[16] Giuliano F, Sanchez-Ramos A, Löchner-Ernst D, Del Popolo G, Cruz N, Leriche A (2007). Efficacy and safety of tadalafil in men with erectile dysfunction following spinal cord injury. Arch Neurol.

[17] Gans WH, Zaslau S, Wheeler S, Galea G, Vapnek jM (2001). Efficacy and safety of oral sildenafil in men with erectile dysfunction and spinal cord injury. J Spinal Cord Med.

[18] Ramos AS, Vidal J, Jauregui M, Barrera M, Recio C, Giner M (2001). Efficacy, safety and predictive factors of therapeutic success with sildenafil for erectile dysfunction in patients with different spinal cord injuries. Spinal Cord.

[19] Ergin S, Gunduz B, Ugurlu H, Sivrioglu K, Oncel S, Gok H (2008). A placebo-controlled, multicenter, randomized, double-blind, flexible-dose, two-way crossover study to evaluate the efficacy and safety of sildenafil in men with traumatic spinal cord injury and erectile dysfunction. J Spinal Cord Med.

[20] Maytom M, Derry F, Dinsmore W, Glass C, Smith M, Orr M (1999). A two-part pilot study of Sildenafil (VIAGRA TM) in men with erectile dysfunction caused by spinal cord injury. Spinal Cord.

[21] Soler J, Previnaire J, Denys P, Chartier-Kastler E (2007). Phosphodiesterase inhibitors in the treatment of erectile dysfunction in spinal cord-injured men. Spinal Cord.

[22] Lombardi G, Macchiarella A, Cecconi F, Del Popolo G (2009). Efficacy and safety of medium and long‐term tadalafil use in spinal cord patients with erectile dysfunction. J Sex Med.

[23] Del Popolo G, Marzi VL, Mondaini N, Lombardi G (2004). Time/duration effectiveness of sildenafil versus tadalafil in the treatment of erectile dysfunction in male spinal cord-injured patients. Spinal Cord.

[24] Giuliano F, Rubio-Aurioles E, Kennelly M, Montorsi F, Kim ED, Finkbeiner AE (2006). Efficacy and safety of vardenafil in men with erectile dysfunction caused by spinal cord injury. Neurology.

[25] Guttmann L, Munro A, Robinson R, Walsh J (1963). Effect of tilting on the cardiovascular responses and plasma catecholamine levels in spinal man. Spinal Cord.

[26] Mathias CJ, Christensen NJ, Corbett JL, Frankel HL, Goodwin TJ, Peart WS (1975). Plasma catecholamines, plasma renin activity and plasma aldosterone in tetraplegic man, horizontal and tilted. Clin Sci Mol Med.

[27] Ethans KD, Casey AR, Schryvers OI, MacNeil BJ (2003). The effects of sildenafil on the cardiovascular response in men with spinal cord injury at or above the sixth thoracic level. J Spinal Cord Med.

[28] Sipski M, Alexander C, Guo X, Gousse A, Zlamal R (2003). Cardiovascular effects of sildenafil in men with SCIs at and above T6. Topics in spinal cord injury. Rehabilitation.

[29] Taylor J, Baldo OB, Storey A, Cartledge J, Eardley I (2009). Differences in side‐effect duration and related bother levels between phosphodiesterase type 5 inhibitors. BJU Int.

[30] Moemen M, Fahmy I, AbdelAal M, Kamel I, Mansour M, Arafa M (2008). Erectile dysfunction in spinal cord-injured men: different treatment options. Int J Impot Res.

